# Gingival-derived mesenchymal stem cell therapy regenerated the radiated salivary glands: functional and histological evidence in murine model

**DOI:** 10.1186/s13287-024-03659-7

**Published:** 2024-02-16

**Authors:** Hagar M. Zayed, Nevine H. Kheir El Din, Ashraf M. Abu-Seida, Asmaa A. Abo Zeid, Ola M. Ezzatt

**Affiliations:** 1 Department of Oral Medicine, Periodontology and Oral Diagnosis, Faculty of Dentistry, Ain Shams University, 20 Organization of African Union St., Cairo, 1156 Egypt; 2 Department of Surgery, Anesthesiology, and Radiology, Faculty of Veterinary Medicine, Cairo University, Cairo, 13736 Egypt; 3 Department of Histology, and Cell Biology, Faculty of Medicine, Ain Shams University, Cairo, 11591 Egypt; 4 Central Lab of Stem Cells and Biomaterial Applied Research (CLSBAR), Faculty of Dentistry, Ain-Shams University, Cairo, Egypt

**Keywords:** Apop-Tag Peroxidase, Anti-proliferating cell nuclear antigen, Oral derived stem cells, Guinea pigs, Xerostomia

## Abstract

**Background:**

Radiotherapy in head and neck cancer management causes degeneration of the salivary glands (SG). This study was designed to determine the potential of gingival mesenchymal stem cells (GMSCs) as a cell-based therapy to regenerate irradiated parotid SG tissues and restore their function using a murine model.

**Methods:**

Cultured isolated cells from gingival tissues of 4 healthy guinea pigs at passage 3 were characterized as GMSCSs using flow cytometry for surface markers and multilineage differentiation capacity. Twenty-one Guinea pigs were equally divided into three groups: *Group I/Test*, received single local irradiation of 15 Gy to the head and neck field followed by intravenous injection of labeled GMSCs, *Group II/Positive control*, which received the same irradiation dose followed by injection of phosphate buffer solution (PBS), and *Group III/Negative control*, received (PBS) injection only. Body weight and salivary flow rate (SFR) were measured at baseline, 11 days, 8-, 13- and 16-weeks post-irradiation. At 16 weeks, parotid glands were harvested for assessment of gland weight and histological and immunohistochemical analysis.

**Results:**

The injected GMSCs homed to degenerated glands, with subsequent restoration of the normal gland histological acinar and tubular structure associated with a significant increase in cell proliferation and reduction in apoptotic activity. Subsequently, a significant increase in body weight and SFR, as well as an increase in gland weight at 16 weeks in comparison with the irradiated non-treated group were observed.

**Conclusion:**

The study provided a new potential therapeutic strategy for the treatment of xerostomia by re-engineering radiated SG using GMSCs.

## Introduction

Radiotherapy plays a crucial role in the curative treatment of the majority of patients with head and neck cancer [1]. However, the damage imposed by radiotherapy to normal tissues surrounding the tumor may cause severe complications. In particular, co-irradiation of the salivary glands results in a progressive loss of gland function that begins early in the course of radiotherapy [2].

Quantitative salivary changes in irradiated patients predispose to oral dryness, and impairment of normal oral functions such as speech, chewing, and swallowing [3]. As well as, frequent ulceration and injury of atrophied oral mucosa [1]. Furthermore, the changes in saliva composition (decreased buffer capacity, pH, and immunoprotein concentrations) may result in rapidly progressing radiation caries [4].

Current therapies for xerostomia involve the administration of artificial saliva substitutes or sialagogues to increase the flow rate of saliva [5]. In addition, parasympathomimetic drugs such as pilocarpine and cevimeline have been used to stimulate residual functional salivary gland tissues [6]. Unfortunately, those used therapies are short-lived and have multiple negative side effects including nausea, diarrhea, and excessive sweating [7]. Furthermore, the current pharmacological interventions failed to prevent post-irradiation salivary gland dysfunction [8]. Therefore, novel therapeutic approaches to restore salivary gland function for these patients were required.

Salivary gland regeneration depending on cell-based therapy has shown the potential to permanently restore salivary gland secretory function [9]. Transplantation of various stem cells, such as intercalated duct cells connecting terminal acini and striated ducts of the salivary gland, c-kit-positive duct cells in the excretory duct of human salivary glands [10], as well as salivary gland-derived progenitor cells isolated from duct-ligated animals and bone marrow-derived stem cells [11–13], adipose-derived stem cells [14], and induced pluripotent stem [15] has been reported to be used for salivary gland tissue regeneration.

Generally, autogenous mesenchymal stem cells have demonstrated promising results as salivary gland regenerative therapy [16, 17]. However, the process of isolation was sometimes technique-sensitive or invasive, besides the short life span and difficulty in isolating autologous stem cells from a severely injured gland [18].

Gingival mesenchymal stem cells (GMSCS) are a subpopulation of mesenchymal stem cells (MSCs) isolated from the lamina propria of gingival connective tissues [19], with notable regenerative properties [20]. Compared to other MSCs, GMSCs are abundant, homogenous, and easily obtainable with a faster proliferation rate [21]. Furthermore, Gingival tissues exhibited scarless wound-healing properties. Interestingly, these cells also display stable phenotypes and telomerase activity in long-term cultures and are not tumorigenic [22]. Investigating the regenerative capacity of GMSCs seems to be crucial for further clinical applications in salivary gland regeneration. However, this approach has been hampered by insufficient published research on this source of stem cell populations [23].

This study attempted to investigate whether GMSCs could restore salivary function, as well as the structure of salivary gland tissues when intravenously injected in a murine model subjected to radiation-induced parotid gland degeneration. Additionally, we also measured the apoptosis and proliferation of salivary cells to explore the mechanisms of possible salivary function restoration as a result of GMSCs transplantation.

## Materials and methods

### Test subjects

This experimental study included twenty-one clinically healthy male guinea pigs weighting about 450–500 g with age ranging from 3–4 months. The subjects were housed in the Faculty of Veterinary Medicine, Cairo University. Animal care and all experimental procedures were conducted by the ARRIVE guidelines (Animal Research: Reporting of In Vivo Experiments) laid down by the National Institute of Health (NIH) (NIH Publication No. 85–23, revised 2011) and by local laws and regulations [24]. The sample size was calculated to be adequate according to the “Resource equation method” where E value = 18 *(E* = *Total number of animals − Total number of groups)* [25]. All subjects received fresh leafy vegetables and pellets as nutrition as well as sterilized water (ad libitum) and their cages were kept in daily clean and conventional conditions at (24 ± 2 °C) with 12-h day/night alternating cycles and closely monitored by [A.S] for any symptoms of severe illness or pain which were considered a humane endpoint for the subject. The study protocol was approved by the Faculty of Dentistry, Ain Shams University, Research Ethics Committee (FDASU-REC ID 091731).

### GMSCs isolation, culturing, and characterization

Four healthy guinea pigs with a range age of 3–4 months were the donors for allogenic GMSCs. The guinea pigs were anesthetized with 2.5 mg/kg Xylazine hydrochloride (HCI) *(Adwia Co., 10th Ramadan City, Egypt)*, and 30 mg/kg Ketamine HCl *(Egyptian International pharmaceutical industries company E.I.P.I.CO, 10th of Ramadan City, Egypt)* given intra-muscular [26]. Gingival tissues were then obtained via gingivectomy techniques of the maxillary and mandibular incisor teeth [19]. The animals were maintained in normal conditions and were fed with a soft diet for an average healing period of one week.

GMSCs isolation, culturing, and expansion techniques were adapted from Zhang et al. [27]. Briefly, the harvested gingival tissues were rinsed with 25 ml sterile Hanks Balanced Salt Solution (HBSS) *(Lonza company, Basel, Switzerland)* and cut into small pieces with the addition of 0.2% collagenase *(SERVA Electrophoresis GmbH, Heidelberg, Germany)* and 0.1% dispase solution *(Sigma-Aldrich GmbH, Hamburg, Germany)*. The tissue fragments were then incubated at 37 °C for two hours. Cell pellets from centrifuged incubated tissues were cultured in T-25 cell culture flasks *(Thermo Fisher Scientific, Massachusetts, USA)* containing Alpha MEM-based medium *(Lonza)*, 10% fetal bovine serum (FBS) *(Life Science Group, Bedford, UK)*, Penicillin/Streptomycin 5 ml *(Lonza)* and Fungizone 500 μl *(Lonza)* as the culture media and incubated in 37°C incubator with 5% CO2. Cells were sub-cultured in larger flasks when they were approximately 90% confluence and the cells were characterized for MSCs identification based on the following: Presence of confluent cells of homogenous morphology of fibroblast-like spindle-shaped plastic adherent cells in monolayer culture after 10 days, the positive expression of CD 90 and CD 105 surface markers and the negative expression of CD 34 and CD 45 surface markers, and in vitro multilineage differentiation potential to functional and matrix-producing osteoblasts, adipocytes, and chondroblasts, which are believed to be the minimal set of standard criteria for MSCs characterization according to the International Society for Cellular Therapy [28].

### Grouping, irradiation, cell labeling and transplantation

Guinea pigs were randomly divided using a computer-generated random table into three different groups, (7 guinea pigs of an age range of 3–4 months) per group as follows: *GroupI [IR/GMSCs]*, for subjects received irradiation immediately followed by GMSCs injection as the test group,*Group II [IR/PBS]*, received irradiation followed by Phosphate-Buffered-Saline (PBS) injection as the positive control group [Potassium chloride (KCl), Potassium phosphate monobasic (KH_2_PO_4_), Sodium chloride (NaCl), Sodium phosphate dibasic (Na_2_HPO_4_•7H_2_O)* (Sigma)*] and *Group III [PBS]* subjects received neither irradiation nor cell transplantation but injected only by PBS and considered the negative control group. The allocation procedure and treatment were performed by [A.S.], while outcome assessment was performed by [H.Z] and treatment administration by [A.A] who were blinded for the type of treatment in each group.

Irradiation-induced parotid gland hyposalivation procedure was elicited by single local head and neck irradiation exposure of 15 Gy using a Gamma cell 220 Cobalt-60 Irradiation Unit *(MDS Nordion, Ottawa, Canada)*, with the rest of the body being shielded with 3 cm of lead to reduce the beam strength to 3% in this area to achieve 30% degeneration of salivary gland [11, 29].

Chloromethyl-Dialkylcarbocyanine (CM-Dil) cell labeling dye *(CellTracker™ CM-Dil, C7000, Invitrogen Life Technologies, Carlsbad, CA)* was prepared using the manufacturer’s instructions. A solution of 1 × 10^6^ GMSCs/ 1ml (PBS) obtained from cell culture passage 3 was prepared and labeled with 2 µL CM-Dil solutions (1 mg/mL) immediately before cell transplantation by injection, subjects in Group I received 500 μL of labeled GMSCs solution through lateral saphenous vein of the hind leg immediately after irradiation, and this injection was repeated weekly for three consecutive weeks according to a previously described method for systemic transplantation of mesenchymal stem cells [30]; similar protocol were applied for control groups using sterile 1ml PBS. All subjects were followed for 16 weeks post-irradiation.

### Functional and morphological assessment of SG

Secretory function of the salivary glands was assessed by the following procedure; Xylazine HCl and 30 mg/kg and 2.5 mg/kg Ketamine HCl intra-muscular injection were followed by whole saliva collection after stimulation of secretion using Pilocarpine *(Sigma)* 0.5 mg /kg body weight administered subcutaneously. Saliva was then obtained from the oral cavity by micropipette through ten minutes period, placed into 1 ml microcentrifuge tubes, and its volume was determined gravimetrically [11].

Salivary flow rate (SFR) (mL/min) and animal body weight (BW) (grams) were assessed for each subject at baseline (the day before radiation), 11 days (early effect), as well as at 8 weeks, 13 weeks and 16 weeks post-irradiation (late effects). At 16 weeks post-irradiation, the guinea pigs were euthanized with an overdose of thiopental sodium injected intracardiac, and their parotid glands were harvested and morphologically assessed for macroscopic changes, and gland weight (GW) (grams) was measured using a sensitive scale [11].

### Cell tracking, histological examination, and immunohistochemical staining

The parotid gland tissues were examined under a confocal laser scanning fluorescent microscope *(Leica, STELLARIS 8, Germany)* 16 days after injection to detect CM-Dil-labeled GMSCs.

The harvested parotid gland tissue samples obtained from each guinea pig (7 in each group) were fixed in 10% (weight/volume) formaldehyde in 0.2 ml PBS, dehydrated in graded ethanol, and embedded in paraffin. For histological examination, each gland sample was deparaffinized and rehydrated, then, three sections of 4-5μm in thickness were obtained and processed for Hematoxylin and Eosin stain (H&E) and other three sections for immunohistochemical staining.

The Apop-Tag Peroxidase In situ apoptosis detection Kit *(Sigma)* was used for immunohistochemical detection of apoptotic cells by detecting DNA cleavage and chromatin condensation associated with apoptosis using a mixed molecular biological–histochemical system. Following the manufacturer’s instructions, the deparaffinized and rehydrated slides were pre-treated with protein digestion enzyme (proteinase) for 15 min at room temperature, and endogenous peroxidase activity was blocked for 10 min by 3% H_2_O_2_ in methanol. Terminal deoxynucleotidyl transferase (TdT) enzyme was incubated with the slides for 1 h and then, anti-digoxigenin conjugated for 30 min at room temperature. Peroxidase substrate was applied to develop the reaction color, and the nuclei of apoptotic cells were stained dark brown.

Anti-proliferating cell nuclear antigen (PCNA) staining was performed with the PCNA staining kit *(Sigma)*. Before antibody labeling, the slides were treated three times with a citrate buffer solution (9 ml of 0.1 M citric acid and 41 ml of 0.1 M sodium citrate in 450 ml distilled water) in a 600W microwave for 5 min and then allowed to cool to room temperature. Thereafter, tissue sections were incubated with primary mouse PCNA antibody overnight at 4 °C. Tissue sections were then washed in PBS, blocked, and incubated with anti-mouse IgG for 1 h at room temperature.

**Fig. 1:**
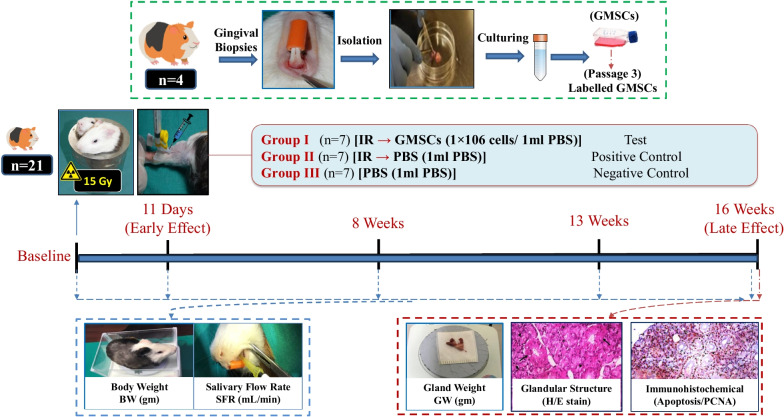
Schematic representation of the experimental design and outcomes

In each immune-stained section, at least, three microscopic fields showing immunopositivity were selected (brown nuclear staining of any intensity was considered positive, and blue nuclear staining was considered negative). Photomicrographs of the selected fields per section were captured using a digital camera *(Olympus, Japan)* mounted on a light microscope *(BX60, Olympus, Japan)*. Digital images at 100× and 400× magnifications were then transformed into the computer to be examined by a blinded investigator for the assessment of the gland’s microscopic structure in (H&E) stained sections. While, for immune-stained sections, the mean area fraction (MAF) of Apop-Tag positive apoptotic cells and PCNA-positive proliferating cells were calculated automatically as the mean percentage of immunopositive area to the total area of the three microscopic field using Image J software *(National Institutes of Health, Bethesda, MD)*. A Schematic representation of the experimental design and outcomes analysis is summarized in **(**Fig. 1**).**

### Statistical analysis

Data management and statistical analysis were performed using the Statistical Package for Social Sciences (SPSS) *(IBM SPSS Statistics for Windows, Version 18.0. Armonk, NY: IBM Corp)*. Parametric data were presented as mean ± standard deviation (SD) values, and comparisons between groups were performed by ANOVA test, while nonparametric numeric variables were compared by Kruskal Wallis test. Friedman’s test was used to study the changes by time in outcomes within each group. *p* values ≤ 0.05 were considered statistically significant.

## Results

**Fig. 2:**
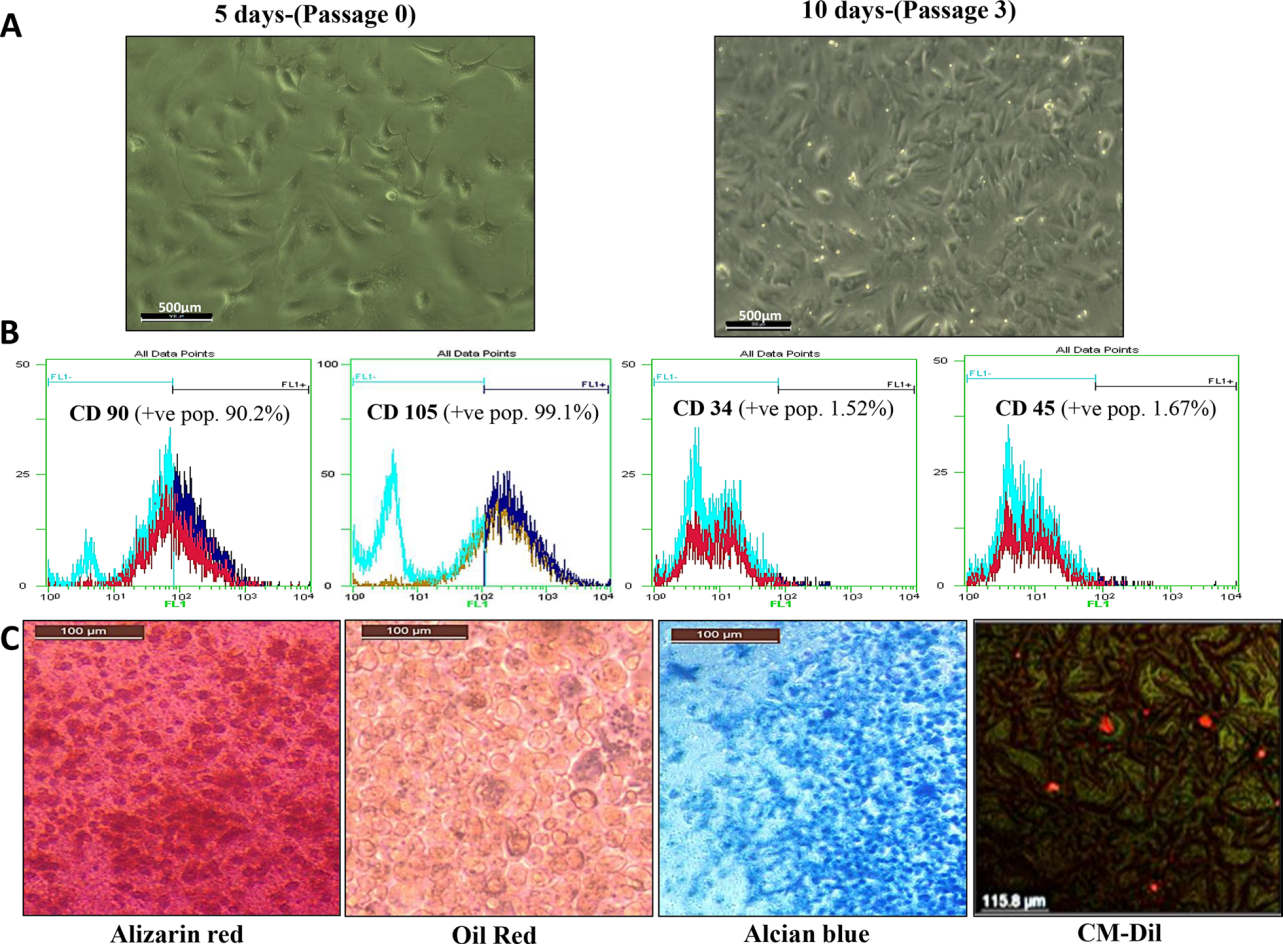
Isolation, characterization, and tracing of GMSCs. **A** Inverted microscope micrographs of five days monolayer primary culture of GMSCs showing fibroblast-like spindle-shaped cells and other cells with star-shaped appearance. While GMSCs after 10 days (passage 3) of culturing showing 90% confluent cells of homogenous morphology of fibroblast-like spindle-shaped cells in monolayer culture. **B** Histogram analysis of cell surface markers of cultured GMSCs (passage 3) determined by flow cytometry showed positive expression of CD 90 and CD 105 surface markers and negative expression of CD 34 and CD 45 surface markers. **C** In vitro multilineage differentiation potential of GMSCs demonstrating osteogenic differentiation of cultured cells confirmed by mineralizing osteocytes stained with Alizarin red. Adipogenic differentiation was confirmed by the presence of lipid droplets stained red with Oil Red stain. Chondrogenic differentiation was confirmed by the presence of chondrocyte lineage cells stained with Alcian blue. The red fluorescence indicated that the CM-Dil-labeled cells were detected and evenly distributed within the differentiated gland tissues after 16 days

Isolated cultured cells were identified by characteristic spindle shape and plastic adherence at 5 days; then, cells were sub-cultured for 10 days showing 90% confluency at passage 3 **(**Fig. 2A**).** Cells from passage 3 were characterized as GMSCSs by being positive for CD 90 and CD 105 surface markers and negative for CD 34 and CD 45 surface markers **(**Fig. 2B**)**, as well as, their multilineage differentiation capacity into osteoblasts, adipocytes and chondrocytes when cultured in appropriate induction media. Moreover, the tracing of intravenously injected GMSCS demonstrated that CM-Dil-positive cells homed and remained in parotid gland tissue for 16 days at the time of gland harvesting **(**Fig. 2C**)**.

### Functional and macromorphological changes of SG

All subjects included in the study completed the experimental protocol with no reported adverse events or dropouts that necessitated termination of the study or scarifying animals for the humane endpoint as monitored and reported by [A.S] on a half-weekly regular basis.

**Fig. 3:**
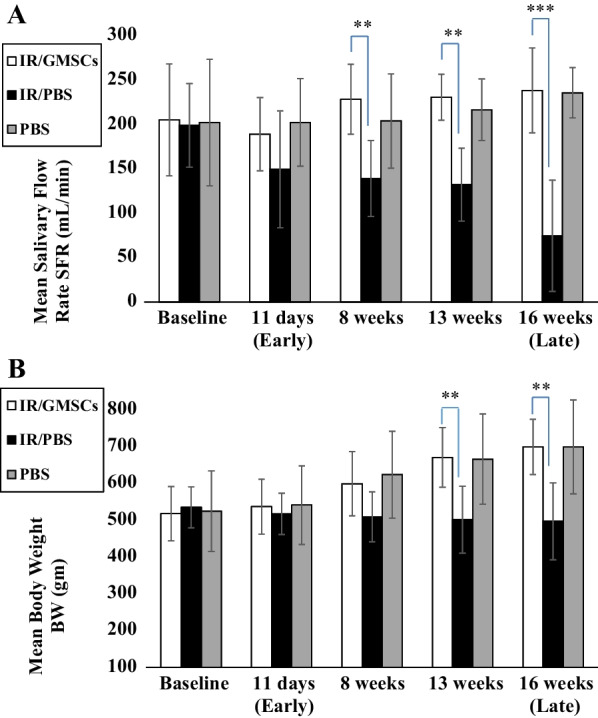
Graphical presentations of functional outcomes. **A** The mean salivary flow rate (SFR, mL/min) and **B** The mean body weight (BW, gm) at 11 days, 8 weeks, 13 weeks, and 16 weeks post-irradiation. **p ≤ 0.01 and *** *p* ≤ 0.001 [IR/GMSCSs] compared to the [IR/PBS] group. Data are expressed as the mean ± SD. n = 7 for each group

The GMSCs-treated subjects showed an initial insignificant reduction in the mean SFR at 11 days (early effect) compared to the baseline levels, then showed a gradual significant increase from (189.29 ± 41.21mL/min) to (238.57 ± 47.93mL/min) at 16 weeks post-irradiation (late effect). The SFR levels in the [IR/GMSCs] group were comparable to those of normal non-irradiated control subjects [PBS] at all intervals. The radiated untreated group [IR/PBS] showed significantly lower mean levels of SFR in comparison with other groups at 8 weeks (*p* = 0.05), 13 weeks (*p* = 0.05) and reached (74.71 ± 62.8mL/min) at 16 weeks post-irradiation (*p* = 0.001) as demonstrated in (Fig. 3A).

Subjects in the [IR/GMSCs] group and those in the [PBS] group showed an increase in mean values of BW in all study intervals, in contrast to subjects in the [IR/PBS] group which showed a decrease in the mean BW early in the 1st eleven days post-irradiation and this reduction was observed at all intervals. The mean BW was statistically significantly different between [IR/GMSCs] and [IR/PBS] groups at 8 and 16 weeks as shown in (Fig. 3B).

**Fig. 4:**
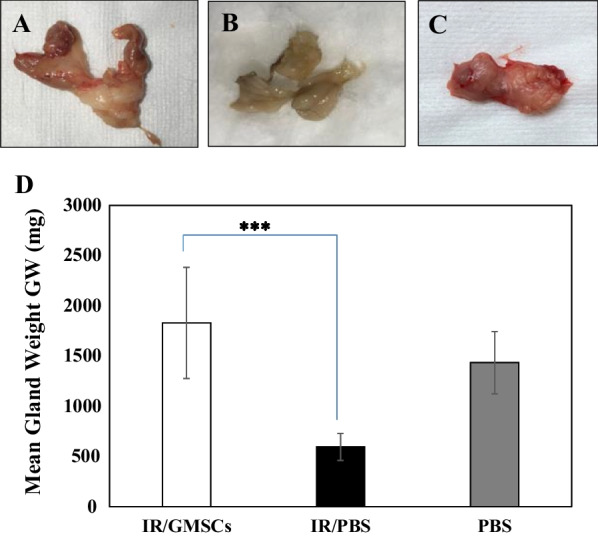
Macromorphological changes and weight of parotid gland. **A** GMSCs-treated group showing normal size and macroscopic structure, **B** Radiated group with smaller and degenerated appearance compared to **C** Control group. **D** Glandular weight (GW, gm) was significantly lower at 16 weeks post-irradiation. *** *p* ≤ 0.001 [IR/GMSCSs] compared to [IR/PBS] group

At 16 weeks post-irradiation the least value of mean Glandular weight (GW) was recorded in the radiated group and was significantly different from both GMSCs-treated and control groups (*p* = 0.000), while no significant difference between the GMSCs-treated glands and the non-irradiated-control-glands (Fig. 4A–D).

### Histomorphological and immunohistochemical changes in the irradiated parotid gland

**Fig. 5:**
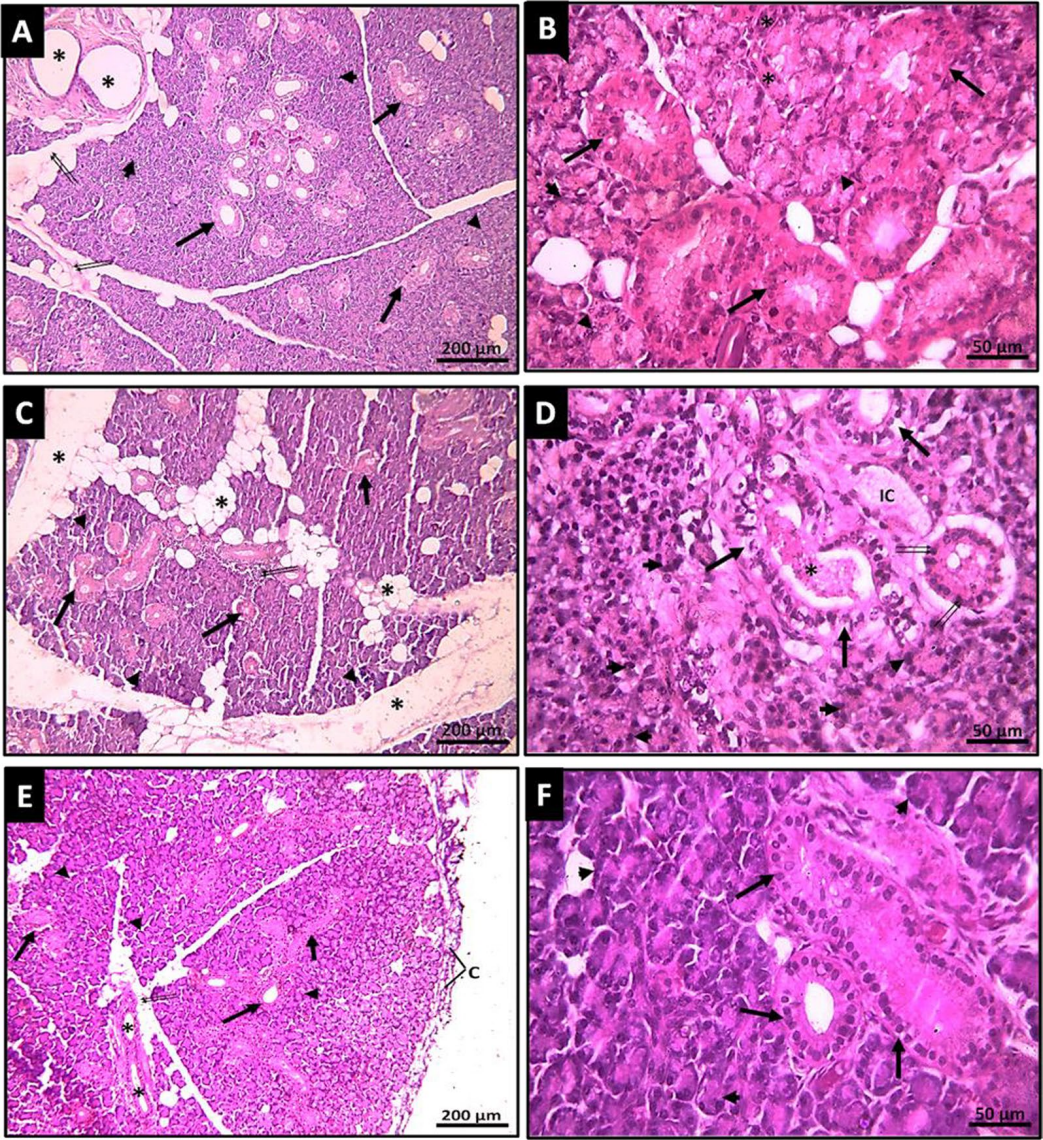
Photomicrographs of H&E-stained sections. **A** The GMSCs-treated parotid gland tissue section with restored normal structural architecture; the interlobular connective tissue is thin with some adipocytes (↑↑) and interlobular excretory ducts (*); each lobule is formed of nearly normal tightly packed serous acini (arrowheads) and numerous striated ducts (↑). (H&E ×100). **B** A higher magnification GMSCs-treated parotid gland tissue section showing closely packed serous acini with restoration of their basal basophilia and apical acidophilic granules (arrowheads); the intercalated ducts can be seen with narrow lumen and lined by simple squamous epithelium (*); the striated ducts have large lumina and lined by simple columnar cells that exhibit basal acidophilic striations (↑). (H&E X400). **C** The radiated parotid tissue section shows a remarkable increase in interlobular space with an apparent increase in adipocytes (*), denoting significant parenchymal atrophy with widely atrophied serous acini (arrowheads) and noticeable inter-acinar spaces (↑↑); the striated ducts exhibit noticeable dilatation (↑). (H&E ×100). **D** Higher magnification of the radiated parotid gland tissue section shows completely disorganized and atrophied serous acini with pyknotic nuclei (arrowheads) and noticeable inter-acinar spaces (↑↑); the striated duct exhibits vacuolated cytoplasm with pyknotic nuclei (↑) and dilated lumen (*). (H&E ×400). **E** The section of normal parotid tissue (control) showing normal structural architecture, surrounded by a capsule (C) arises from its loose interlobular connective tissue with some adipocytes (↑↑) and interlobular excretory ducts (*); each lobule is formed of tightly packed serous acini (arrowheads) and numerous striated ducts (↑). (H&E ×100). **F** A higher magnification of normal parotid tissue section (control) shows closely packed serous acini with basal basophilia and apical acidophilic granules (arrowheads), the striated ducts have large lumina and are lined by simple columnar cells with basal acidophilic striations (↑). (H&E ×400)

Photomicrographs of H-E-stained sections of parotid gland tissues in the GMSCs-treated group revealed restoration of gland normal histological acinar and tubular structure that was comparable to the tissues from the control group. On the other hand, the irradiated group showed marked degeneration of the salivary gland tissue as shown in (Fig. 5A–F).

**Fig. 6:**
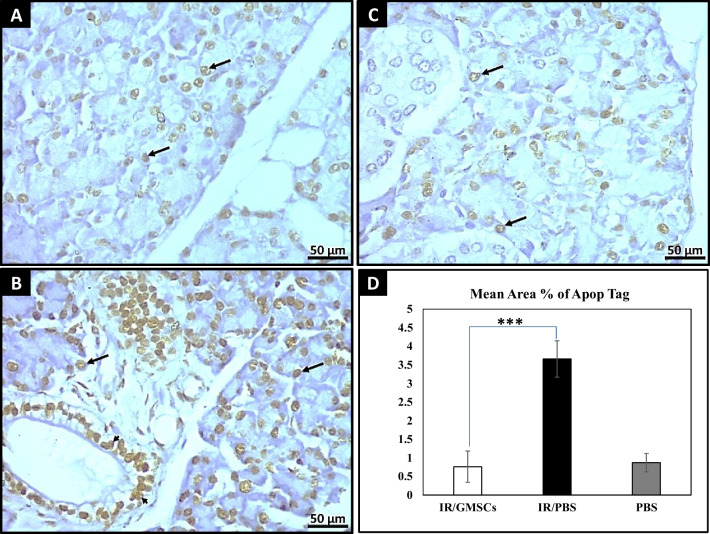
Apop-Tag peroxidase immunoreaction. **A** GMSCs-treated immune-stained section showing apparent low expression of positive apoptotic nuclei of the acinar cells (↑) after irradiation. (Apop Tag, ×400). **B** The immune-stained section of the radiated parotid gland shows high expression of positive apoptotic nuclei in both the acinar cells (↑) and the ductal cells (arrowheads). (Apop Tag, ×400). **C** The immune-stained section of normal control parotid salivary gland tissues shows the expression of positive apoptotic nuclei in the acinar cells (↑). (Apop Tag, ×400). **D** A bar graph showing a significantly higher mean area percentage of Apop-Tag immune positive reaction was recorded at 16 weeks post-irradiation, while there was no significant difference between the GMSCs-treated group and the control group. *** *p* ≤ 0.001 [IR/GMSCSs] compared to [IR/PBS] group

**Fig. 7:**
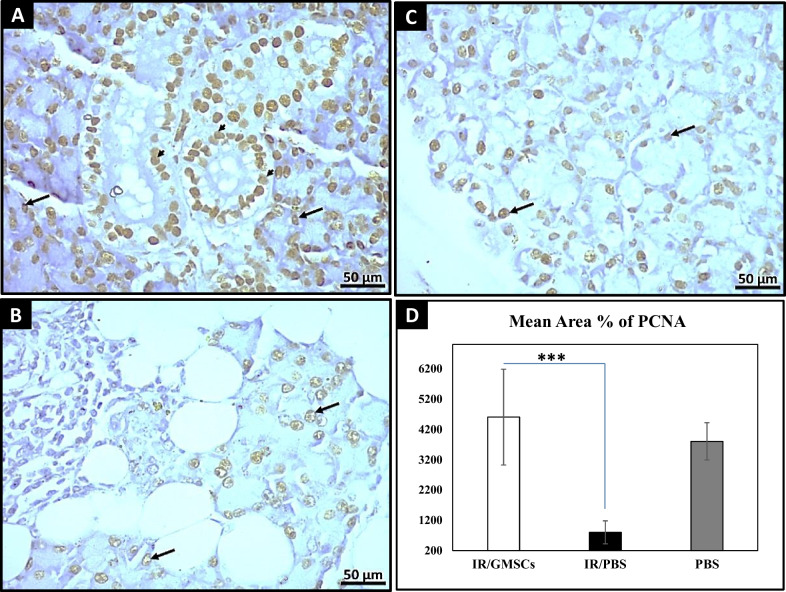
PCNA immunoreaction.**A** GMSCs-treated immune-stained section showing apparent high expression of positive PCNA nuclei of the ductal cells (arrowheads) as well as, acinar cells (↑) (PCNA, ×400). **B** The immune-stained section of the radiated parotid gland shows a low expression of PCNA-positive nuclei in the acinar cells (↑). (PCNA, ×400) **C** The immune-stained section of normal control parotid salivary gland tissues shows the high expression of PCNA-positive nuclei in the acinar cells (↑). **D** A bar graph showing that the GMSCs-treated group and Control group significantly recorded higher values of mean area percentage of PCNA immune positive reaction, while there was no significant difference between the stem cell group and the control group. *** *p* ≤ 0.001 [IR/GMSCSs] compared to [IR/PBS] group

A significantly higher MAF of Apop-Tag positive apoptotic cells was recorded at 16 weeks post-irradiation in immune-stained sections in the irradiated group compared with GMSCs-treated (*p* = 0.00) (Fig. 6A–D). Moreover, sections in the GMSCs-treated group significantly recorded the highest MAF (*p* = 0.00), as well as the number (*p* = 0.001) of PCNA-positive proliferating cells compared to the radiation group as shown in (Fig. 7A–D). Moreover, the Post hoc test revealed no significant difference between the GMSCs-treated group and the control group in MAF of Apop-Tag positive apoptotic cells, number, and MAF of PCNA-positive proliferating cells.

## Discussion

This study showed that the cell therapy approach through intravenous transplantation of GMSCs had regenerated irradiation-damaged salivary gland tissue and rescued their function; as demonstrated by restoration of saliva production, animal body weight, gland weight and promotion of gland tissue regenerative activity.

The gingival tissue is an accessible and easy autogenous source to isolate GMSCs, with no need for highly equipped operating rooms or a specific local environment, systemic patient inclusion criteria, or ethical consideration when compared to previously investigated other sources of MSCs for irradiated salivary gland regeneration, such as embryonic, adipose or bone marrow tissues. Furthermore, studies have demonstrated their potent immunomodulatory/anti-inflammatory effects and regenerative therapeutic potentials in a variety of preclinical models of human diseases and craniofacial defects. GMSCs were well tolerated by different preclinical recipient hosts without any systemic adverse effects, such as immunological reactions, toxicity, or carcinogenesis [31, 32]. The previous data justified the rationale of the current study in advancing research in the field of salivary gland regeneration.

The protocol of isolation and characterization of the GMSCs in the current study was chosen because it was already documented that a purified population of GMSCs could be obtained in the first passage (about 2 weeks after the initiation of culture) using this protocol, without additional growth factors to avoid any modification in protein synthesis and intracellular trafficking that will indirectly affect the biological behaviors of GMSCs [27], and this would justify the future clinical applications. CM-Dil fluorescent dye was selected for the in vivo labeling of GMSCs, because of its documented properties, including its faster loading time, ease of use, and lack of impact on colony formation, proliferation, and multi-differentiation potential of labeled cells [33].

The studies on different animal models using different radiation doses, either single or fractionated have been reviewed and reported that 15–20 Gy would result in about 30–40% reduction in the salivary flow rate [29]. Thus, to achieve about 30% radiation-induced degeneration of the parotid gland during head and neck irradiation without affecting the general health of the animal, we selected the single irradiation dose of 15Gy.

The regenerative potential of GMSCs in irradiated parotid salivary gland was evaluated using guinea pigs’ animal model, taking into consideration that the parotid gland is reported to be more radiosensitive when compared to the submandibular gland, due to the predominance of the serous cells as reported by Grundmann et al. [29]. Moreover, guinea pig shares similarities with humans regarding hormonal and immunologic responses [34], as well as, histological criteria of the salivary glands [35] which justified using it as an excellent preclinical experimental model in this study.

Interestingly, Tran et al. have demonstrated that no significant difference between systemic (I.V.) and local delivery route (intraglandular injection) for MSCs to repair irradiated SGs regarding SFRs, gland weights, cell proliferation rate, amount of acinar cells and blood vessels and suggested that this might be attributed to homing and paracrine properties of MSCs [12]. The same finding was observed in previously reviewed studies on MSCs of different tissue origin as a cell therapy for radiated SGs [17]. Thus, we choose the intravenous injection for stem cell transplantation which is easier and safer precluding the possible local effect of the injection procedure on the gland and facilitating repeated injection when compared to intraglandular injection.

GMSCs-treated guinea pigs showed an initial reduction in salivary flow rate early at 11 days post-irradiation, while increased lately at 8-, 13- and 16-weeks post-irradiation. These results were consistent with Sumita et al. [36] who demonstrated that bone marrow stem cell-transplanted mice at week 8 and 24 post-irradiation had higher SFR when compared to non-transplanted mice, although SFR decreased at 16 weeks. These results were also by Lin et al. [37] who reported that bone marrow stem cells and differentiated acinar-like cells significantly increased saliva production (at day 55) when transplanted into radiation-treated mice. Moreover, Lim et al. in two former studies [11, 30] reported that the irradiated mice that received stem cells showed a significant increase in the SFR at 12 weeks post-irradiation compared with the irradiated control group.

The SFR results were also consistent with Tran et al. [12] and Jeong et al. [38]. The former reported that at week 8 post-irradiation, bone marrow stem cell-treated mice had their SFR restored to normal levels when compared with the irradiated mice without treatment, while the later showed that stem cells treated glands had a significant increase in the SFR at 8 weeks post-irradiation.

Comparable results were reported by Xiong et al. [39] who showed that subcutaneously injected human adipose stem cells in the irradiated submandibular salivary gland of rats had resulted in a significant increase in SFR compared with the untreated irradiated gland at 24 weeks. Interestingly, our study reported about a 20.6% increase in SFR at 8 weeks and a 26.12% increase at 16 weeks in GMSCs-treated group. While, in a clinical study done by Grønhøj et al.[40] intraglandular injection of adipose stem cells in the human submandibular salivary gland following neck radiotherapy resulted in a significant increase of unstimulated SFR by 33% at 4 weeks and 50% at 16 weeks which was also higher than that observed in the placebo group. The discrepancies in results could be attributed to different routes of cell transplantation.

Urek et al. [41] speculated that irradiation-induced sublethal-DNA damage became apparent during the late-effect phase post-irradiation due to a slow cell turnover rate. Furthermore, Li et al. [42] suggested that chronic effects of radiation may be the consequence of acute damage to salivary glands and chronically affected individuals continued to display significant decreases in SFR for several months or years following radiotherapy. This explains the changes observed in function in our study and is reflected by the results of SFR.

The effect of irradiation and GMSCs treatment on general health was detected by a significant decrease in the BW of the radiation group in comparison with the GMSCs-treated group at 11 days post-irradiation which showed a significant increase in BW and was almost similar to the increase observed in normal control group.

The early effect of irradiation on BW has not been investigated thoroughly in the published literature; however, it was reported that there was an impairment in the gland tissue and reduced secretion of certain components of normal saliva at the early phase of irradiation [43], which may be reflected on the general health and the BW of the irradiated animals thus could explain the results obtained in this study. Comparable results were also reported by Jeong et al. [38] who conducted a study on human salivary glands stem cells to ameliorate the hyposalivation of irradiated rat salivary gland, they reported that the BW of irradiated rats decreased gradually until day 7, while in the stem cells, transplanted group the BW increased to a greater extent compared to the irradiated untreated group.

The radiation group showed the lowest BW in comparison with the other two groups at 16 weeks post-irradiation, with a continuous reduction in the BW compared to further and significant BW increase observed in the stem cell group. These results were consistent with that of Lin et al. [37] which reported that the total BW of irradiated mice without cell therapy decreased significantly at 55 days post-irradiation compared with bone marrow and acinar-like cell-treated groups.

On the other hand, Lim et al. [11] reported that BW following intraglandular injection of bone marrow stem cells in irradiated submandibular salivary glands of mice were not significantly different compared with irradiated mice at 12 weeks post-irradiation. Interestingly, another study in 2013 done by Lim et al. [30] on intravenously injected human adipose tissue-derived mesenchymal stem cells for the regeneration of irradiated mice submandibular salivary gland, reported that 12 weeks after irradiation there was an increase in the BW in the treated group compared to the irradiated group, although this increase was not statistically significant. The controversy in results could be attributed to the difference in route of injection, type of the irradiated gland, type and regenerative potential of the injected stem cells.

In the current study, the GMSCs-treated group revealed a significant increase in the parotid gland weight when compared to the irradiated non-treated group at 16 weeks post-irradiation. The previous results were from the studies reviewed by Tanaka and Mishima [44]. These results could be attributed to the regenerative effect of the transplanted GMSCs that prevent acinar cell loss post-irradiation compared to the atrophy of the irradiated untreated gland in Group II. On the other hand, the results of the current study were in contrast to Lim et al. [30], who reported that the salivary gland weight at 12 weeks was not significant in the stem cell-treated group compared with the non-treated one. This difference may be attributed to the use of adipose tissue-derived mesenchymal stem cells in that study.

The GMSCs-treated group recorded lower apoptotic activity, higher mean value of area percentage of newly formed tissues, and number of proliferating cells compared to Group II. These results were by An et al. [45] study which reported that systemically infused human adipose mesenchymal stem cells secretome remodel and restore the function of the irradiated submandibular salivary gland in mice through cytoprotection of salivary gland parenchymal, endothelial and progenitor cells by inhibition of apoptosis, and that salivary epithelial (AQP-5), endothelial (CD31), myoepithelial (α-SMA) and salivary gland progenitor cells (c-Kit) were successfully protected from radiation damage and remodeled. Interestingly, Kim et al. [31] have recently reviewed experimental studies assessing the regenerative potential of GMSCs and documented that GMSCs had markedly promoted the regeneration of ductal, acinar, and myoepithelial cells of dissected non-radiated submandibular salivary glands [46].

The regenerative potential of GMSCs is demonstrated as restoration of the damaged tissues probably could be explained via several mechanisms; one of the mechanisms is modulation of the local inflammatory response that was reported by Linard et al. [47]. Similarly, Hong et al. [48] reported that GMSCs significantly reduced the expression of M1-related pro-inflammatory cytokines of TNF-α, IL-6, IL-1β, and M1 markers CD86 and HLA-DR, while moderately increasing the expression of M2-related CD206 and the secretion of anti-inflammatory cytokine IL-10. Moreover, Kim et al. [31] and Negi and Griffin [49] reported that GMSCs promote the generation of regulatory T-cells through the production of anti-inflammatory cytokines such as IL-10, TGF-β1 and showed a suppressive effect on pro-inflammatory Th1 and Th17, thus contributing to immune homeostasis.

The release of paracrine mediators such as growth factors and chemokines was reported to provide acute radioprotection [50]. Thus, the reduction in apoptosis observed in the GMSCs-treated group was probably related to the anti-apoptotic effects of stem cells due to the local paracrine secretion GMSCs-derived bioactive components that reduce parotid gland tissue fibrosis by down-regulating inflammatory factor levels. Moreover, GMSCs expressed high levels of reactive oxygen species, hypoxia-inducible factor (-1 and -2a), superoxide dismutase-2, and manganese superoxide dismutase, which improved their resistance to oxidative stress-induced apoptosis [51]. Thus, we suggested that GMSCs interacted with resident cells and the local environment in response to tissue damage to produce paracrine mediators and growth and proliferative factors which probably explain higher glandular weight and mean area % of PCNA compared to the control PBS group which was injected by basic formula of PBS without calcium or magnesium to avoid the possible effects of these minerals on normal cells.

The study design and outcomes were focused on functional and histological assessment of the treated glands. However, the study was limited by the lack of evidence for direct trans-differentiation of transplanted GMSCs into acinar and ductal cells as proved by Lim et al. regarding allogenic systemically transplanted adipose-derived MSCs [30]. Moreover, the possible heterogeneous mesenchymal stem/stromal cell populations cannot be denied and the detailed robust matrix of functional assays to demonstrate the isolated cells' properties are recommended for further investigations according to the International Society for Cell and Gene Therapy (ISCT) Mesenchymal Stromal Cell committee position statement in 2019 [52].

There was documentation that systemically injected cells have been homed and engrafted into damaged SG tissues. In addition to signs of restored salivary gland function and glandular mass, with provided histological evidence of structural recovery of damaged parotid tissues compared to the irradiated non-treated group. Suggesting that these MSCs either transdifferentiated into acinar and ductal cells or stimulated differentiation of remaining salivary tissue progenitor cells.

## Conclusion

The present study provided for the first-time direct evidence that systemic transplantation of GMSCS in irradiated animals restored salivary gland function and alleviated irradiation-induced xerostomia which subsequently reflected in treated subjects’ general health. This could be possibly attributed to their ability to inhibit apoptosis and promote regeneration of new healthy glandular and ductal cells, thus re-establishing the normal gland architecture. Generally, much more work is required to establish the pathway for clinical research of GMSCs, which involves the determination of suitable donors, the development of standard operating protocols for cell isolation, expansion, storage, transportation, and priming GMSCs before administration, as well as the selection of suitable patient populations for treatment [32, 53]. Moreover, investigating the optimum concentration and timing of transplanted GMSCs to improve the regeneration of the damaged structures of radiated salivary glands at different radiation doses during the late effect of radiation is recommended.

## Data Availability

The original data are available at the corresponding author upon request.

## Abbreviations

ARRIVE: Animal research reporting of in vivo experiments
BW: Body weight
FBS: Fetal bovine serum
GMSCs: Gingival mesenchymal stem cells
GW: Glandular weight
HBSS: Hanks balanced salt solution
H&E: Hematoxylin and Eosin stain
HCI: Hydrochloride
MAF: Mean area fraction
MSCs: Mesenchymal stem cells
NIH: National institute of health
SG: Salivary glands
PBS: Phosphate buffer solution
PCNA: Anti-proliferating cell nuclear antigen
SFR: Salivary flow rate
SPSS: Statistical package for social sciences
TdT: Terminal deoxynucleotidyl transferase
